# Primary hyperparathyroidism due to dual parathyroid adenomas: one orthotopic, one intrathymic in ectopic cervical thymus

**DOI:** 10.1210/jcemcr/luag061

**Published:** 2026-04-22

**Authors:** Migdalia Iglesias, Jason Lewis, Jeffrey Janus, Ejigayehu Abate, Ana-Maria Chindris

**Affiliations:** Clinical Research Unit, Mayo Clinic Florida, Jacksonville, FL 32224, USA; Florida State University School of Medicine, Tallahassee, FL 32306, USA; Department of Pathology, Mayo Clinic Florida, Jacksonville, FL 32224, USA; Department of Otorhinolaryngology, Mayo Clinic Florida, Jacksonville, FL 32224, USA; Department of Internal Medicine, Division of Endocrinology, Mayo Clinic Florida, Jacksonville, FL 32224, USA; Department of Internal Medicine, Division of Endocrinology, Mayo Clinic Florida, Jacksonville, FL 32224, USA

**Keywords:** parathyroid adenoma, ectopic thymus, parathyroid hormone, primary hyperparathyroidism

## Abstract

Primary hyperparathyroidism is most commonly caused by a single parathyroid adenoma. Multiple and ectopic adenomas are less common and can present diagnostic challenges. We present the case of a 57-year-old woman with hypercalcemia, hypercalciuria, and inappropriately normal parathyroid hormone (PTH) levels. Sestamibi scan did not demonstrate abnormal uptake; however, contrast-enhanced 4-dimensional computed tomography imaging reported 2 candidate lesions described as tubular shaped structures located posterior to the inferior pole of each thyroid lobe. Surgical exploration was performed and the surgical pathology reported hypercellular parathyroid tissue resected from the left inferior thyroid bed and benign thymic tissue with embedded hypercellular parathyroid tissue resected from the right inferior thyroid bed. Postoperative calcium levels normalized, and the patient remained eucalcemic at 1-year follow-up. This case underscores the importance of embryologic understanding and thorough surgical exploration in patients with biochemically confirmed primary hyperparathyroidism and inconclusive imaging.

## Introduction

Primary hyperparathyroidism (PHPT) presents with hypercalcemia and elevated or inappropriately normal serum parathyroid hormone (PTH). Some patients may have only mild, asymptomatic hypercalcemia, whereas others also have clinical manifestations including osteopenia or osteoporosis, vertebral fractures, and nephrolithiasis [1]. A single parathyroid adenoma is the most common cause of PHPT, being present in 80% of patients. Although parathyroid adenomas most commonly arise in the inferior parathyroid glands, they can also be found in unexpected ectopic locations because of embryonal migration patterns [1]. The third pharyngeal pouch gives rise to both the inferior parathyroid glands and the thymus. The thymus descends caudomedially to the anterior mediastinum, whereas the inferior parathyroid glands follow the thymus' path to their destination posterior to the thyroid. The inferior glands' longer course to reach their anatomical destination leads to their higher probability of becoming ectopic. The thymus can also become ectopic in the cervical region due to incomplete descent. Ectopic inferior parathyroid glands are most commonly present within the thymus, anterior mediastinum, or thyroid [2, 3]. Ectopic parathyroid adenomas are found in 6% to 16% of adult cases, with one third of these being in the thymus [4]. Although multiple parathyroid adenomas occur in approximately 10% to 15% of cases [5], the occurrence of multiple adenomas comprising both orthotopic and ectopic locations is rare in the literature and its true prevalence remains unknown [1]. Furthermore, to date, there have been no reported cases of an orthotopic parathyroid adenoma occurring simultaneously with an intrathymic adenoma within ectopic thymic issue.

We report a case of PHPT due to 2 parathyroid adenomas with 1 located in the left inferior thyroid bed and the other in ectopic thymic tissue embedded within the right inferior thyroid bed.

## Case presentation

A 57-year-old female presented to the endocrinology department after being referred for a 2-year history of persistent hypercalcemia, noted on routine laboratory testing.

Additional evaluation revealed a nonsuppressed serum PTH, and a 24-hour urine sample demonstrated hypercalciuria (Table 1). Review of systems was positive for fatigue and night sweats. She denied history of nephrolithiasis or fragility fractures. The patient's medical history included hyperlipidemia, hypertension, and a history of breast cancer diagnosed 1 year prior, for which she underwent a lumpectomy and was declared free of disease at the most recent follow up. Her family history was positive for osteoporosis and hypertension in her mother and skin and colon cancer in her father. Notably, there was no family history of calcium abnormalities or parathyroid disorders. Medications included ezetimibe 10 mg daily and metoprolol 25 mg daily, but no drugs known to cause hypercalcemia such as thiazides or lithium, or hypercalciuria such as furosemide or acetazolamide. She was not taking any over-the-counter supplements, specifically no high-dose biotin, known to interfere with the PTH assay. Dietary history revealed adequate calcium consumption. The physical examination was unremarkable. Laboratory test results are presented in Table 1.

**Table 1:** Laboratory testing at the initial presentation, 3-month observation, and 1-year postparathyroidectomy

Laboratory test	Initial presentation conventional units (SI units)	3-month observation conventional units (SI units)	1-year postsurgery conventional units (SI units)	Normal reference range conventional units (SI units)
Total serum calcium (corrected)	10.6 mg/dL (2.65 mmol/L)	10.5 mg/dL (2.63 mmol/L)	9.5 mg/dL (2.38 mmol/L)	8.6-10.0 mg/dL (2.15-2.5 mmol/L)
Ionized serum calcium	5.5 mg/dL (1.38 mmol/L)	ND	ND	4.7-5.4 mg/dL (1.18-1.35 mmol/L)
Serum phosphorus	3.3 mg/dL (1.07 mmol/L)	3.5 mg/dL (1.13 mmol/L)	ND	2.5-4.5 mg/dL (0.81-1.45 mmol/L)
Serum creatinine	0.8 mg/dL (70.72 μmol/L)	0.6 mg/dL (53.04 μmol/L)	0.62 mg/dL (54.81 μmol/L)	0.59-1.04 mg/dL (52.16-91.94 μmol/L)
25 hydroxy vitamin D	28 ng/mL (68.89 nmol/L)	35 ng/mL (87.36 nmol/L)	ND	20-50 ng/mL (49.92-124.80 nmol/L)
1,25 dihydroxyvitamin D	ND	34 pg/mL (81.60 pmol/L)	ND	18-78 pg/mL (43.2-187.2 pmol/L)
Serum parathyroid hormone	27 pg/mL (27 ng/L)	23 pg/mL (23 ng/L)	ND	15-65 pg/mL (15-65 ng/L)
Calcium urine, 24-hour	399 mg/24 hours (9.96 mmol/24 hours)	336 mg/24 hours (8.38 mmol/24 hours)	ND	< 200 mg/24 hours (< 5 mmol/24 hours)
Creatinine, 24-hour	1121 mg/24 (9905 µmol/24 hours)	866 mg/24 (7652 µmol/24 hours)	ND	603-1783 mg/24 hours (5330–15 760 µmol/24 hours)
24-hour urine volume (L)	2.1 L	2.4 L	ND	

Abbreviation: ND, no data.

Because of the mild laboratory abnormalities and absence of definitive symptoms, the initial decision was for short-interval observation with repeat testing.

## Diagnostic assessment

At 3 months’ follow-up, laboratory testing confirmed hypercalcemia with hypercalciuria of 336 mg/24 hours (>250 mg/24 hours [SI: > 6.25 mmol/24 hours]) (reference range < 200 mg/24 hours [SI: < 5 mmol/24 hours]) and inappropriately normal serum PTH (Table 1), strongly suggestive of PHPT. The patient was recommended surgical intervention [6].

**Figure 1:**
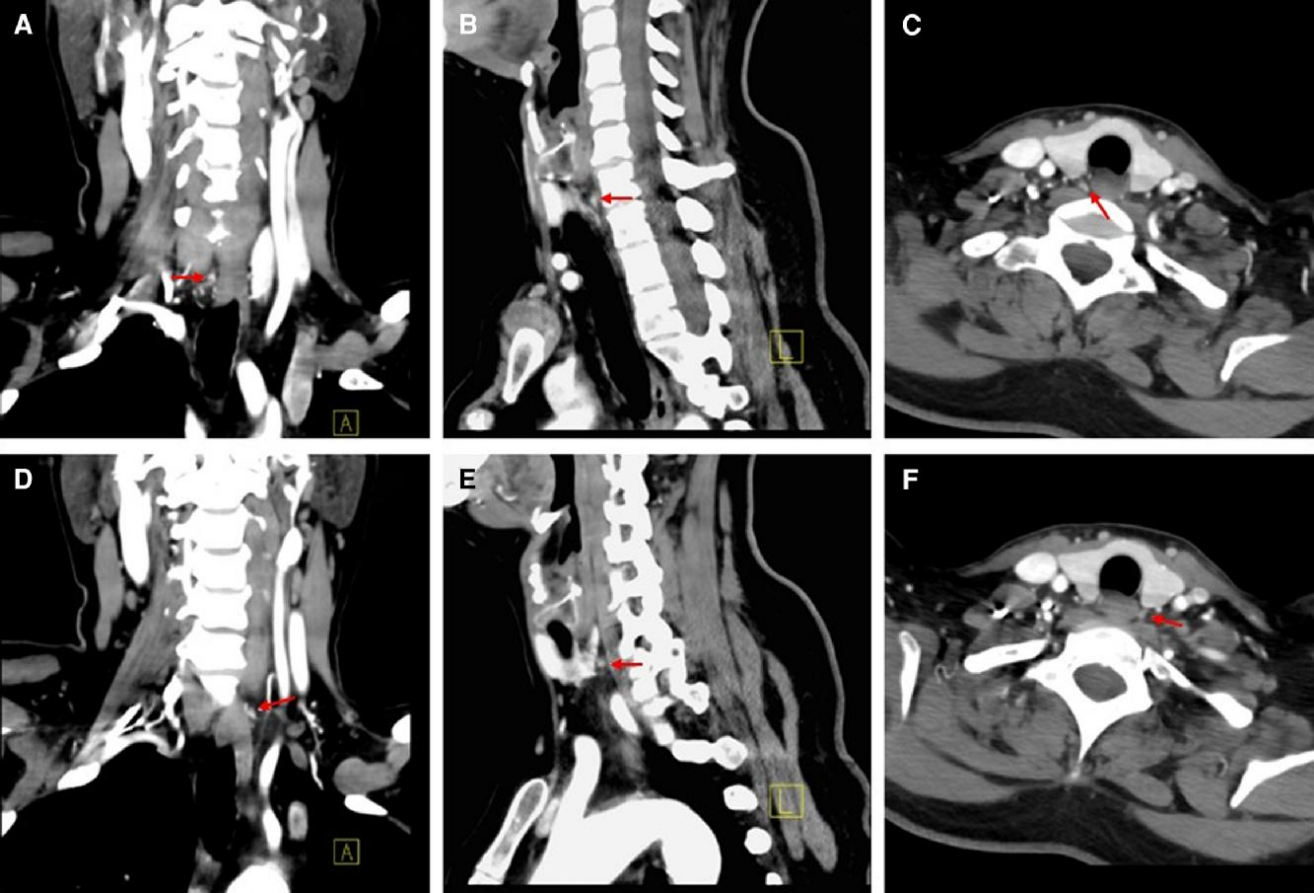
Contrast-enhanced 4-dimensional computed tomography scan showing a 0.5 × 0.2 × 0.3-cm lesion posterior to the inferior pole of the right thyroid lobe in coronal (A), sagittal (B), and transverse (C) views and 1 of similar dimensions posterior to the inferior pole of the left thyroid lobe in coronal (D), sagittal (E), and transverse (F) views.

Localizing studies were performed as the next step. A neck ultrasound did not identify parathyroid adenomas; therefore, a nuclear medicine parathyroid scan with single-photon emission computed tomography/computed tomography (SPECT/CT) and intravenous technetium (Tc) 99 m sestamibi was obtained. This did not show radiotracer avid parathyroid lesions in the neck or upper mediastinum. A contrast-enhanced 4-dimensional (4D) CT scan of the neck demonstrated 2 candidate lesions located posterior to the inferior pole of the right and left pole of the thyroid gland respectively (Fig. 1 A-F).

## Treatment

Given the 4D CT findings, the patient agreed to 4-gland exploration. The procedure was initiated in the standard fashion by making our way through the skin and subcutaneous soft tissues, mobilizing the thyroid, identifying the recurrently laryngeal nerve (on each side) in the tracheoesophageal groove, and mindfully following the nerve superiorly to the interface with the inferior thyroid artery. During this process, the middle thyroid vein was ligated. By then carefully tracing the inferior thyroid artery blood supply, the parathyroid glands on each side were identified. In the left inferior thyroid bed, there were 3 notable findings: (1) a small pedunculated mass confirmed as benign thyroid tissue by frozen sections; (2) inflamed, discolored lymph nodes; and (3) what appeared to be an abnormally enlarged and slightly discolored gland suspicious for left inferior parathyroid adenoma. On the right side, there was thymic tissue with a ball-valving pink mass inside, suspicious for intrathymic parathyroid. The 2 superior parathyroid glands were identified and demonstrated normal color, contour, and consistency, and were thus preserved. The case was somewhat less straightforward as the patient had several pink enlarged lymph nodes in the central compartment that could have been mistaken for parathyroid tissue. Intraoperative PTH, collected from the internal jugular vein, decreased from a baseline of 45.8 pg/mL (SI: 45.8 ng/L) (reference range not applicable for intraoperative PTH) to 7.3 pg/mL (SI: 7.3 ng/L), 33 minutes after removal of the last specimen. Because the PTH value decreased by more than 50%, which is considered a criterion for successful parathyroidectomy, the procedure was concluded [7].

**Figure 2:**
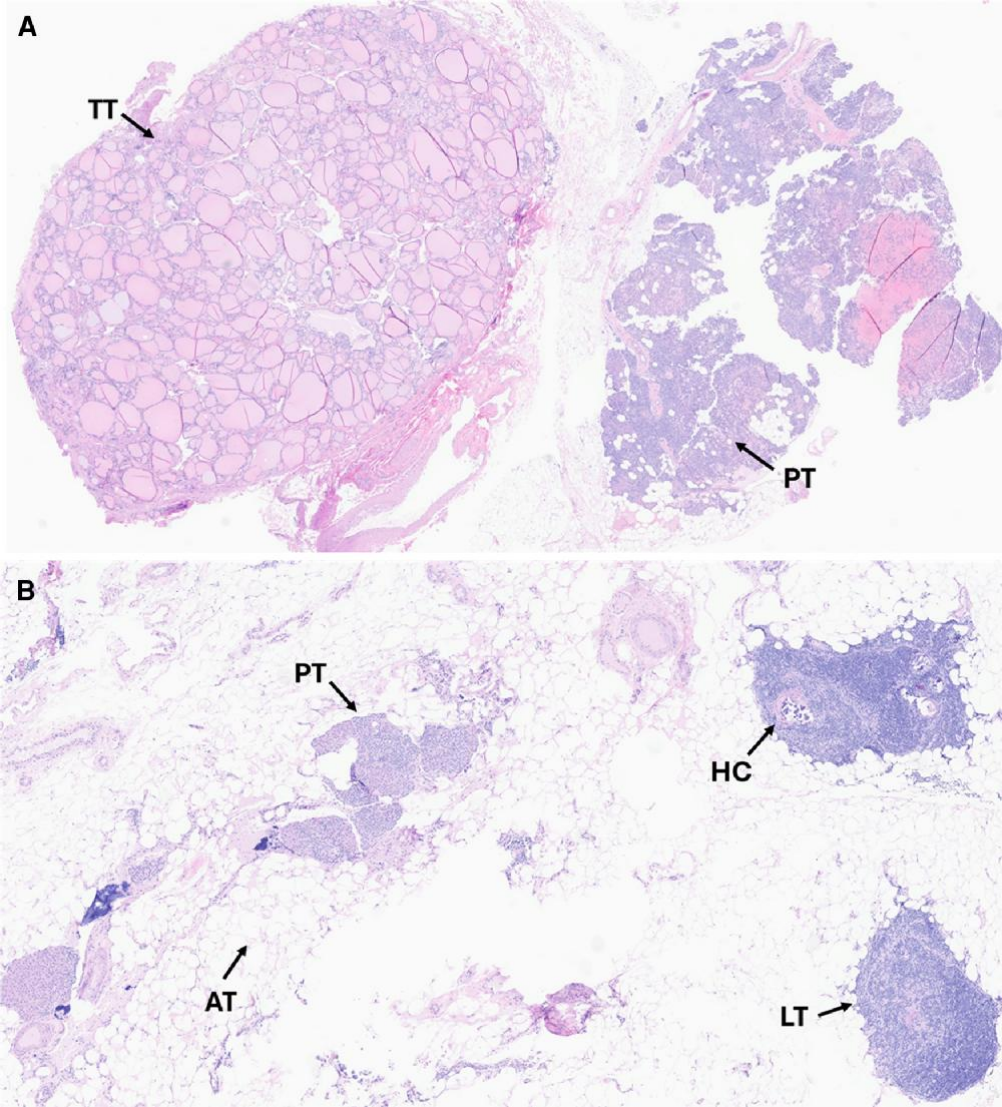
(A): Low-power hematoxylin and eosin image (×20) of a hypercellular parathyroid gland (PT) adjacent to benign thyroid tissue (TT). (B) Medium-power hematoxylin and eosin image (×50) of PT embedded within thymic tissue, the latter characterized by Hassall corpuscles (HC), lymphoid tissue (LT), and adipose tissue (AT).

A total of 5 surgical specimens were sent off. Histopathology reported a left inferior benign thyroid nodule measuring 0.7 cm and a left inferior hypercellular parathyroid gland measuring 0.8 cm and weighing 240 mg (Fig. 2A). The third left inferior specimen consisted of lymphoid tissue. A fourth, right inferior specimen, was also consistent with benign lymphoid tissue and the fifth specimen from the right inferior thyroid bed, measuring 1.4 cm and weighing 500 mg, was reported as hypercellular parathyroid embedded in thymic tissue (Fig. 2B).

## Outcome and follow-up

The postoperative recovery was uneventful, with no symptoms of hypocalcemia. Ten days postoperatively, the serum PTH level was borderline-low at 11 pg/mL (SI: 11 ng/L) (reference range 15-65 pg/mL [SI: 15-65 ng/L]) and total serum calcium level normalized to 9.4 mg/dL (SI: 2.35 mmol/L) (reference range 8.6-10.0 mg/dL [SI: 2.15-2.5 mmol/L]). The patient has remained normocalcemic and asymptomatic 1 year after the surgical intervention.

## Discussion

The understanding of the clinical implications of PHPT has deepened over the past decades, leading to the recognition of 3 main phenotypes: PHPT with mild asymptomatic hypercalcemia, normo-calcemic PHPT, and PHPT with renal and/or skeletal involvement, such as in the case of our patient [5]. Following the hypercalcemia diagnosis in our patient, a PTH level was obtained and found inappropriately normal for the serum calcium levels. The onset, momentum, and severity of hypercalcemia, usually less than 12 mg/dL (SI: 3 mmol/L) along with the unsuppressed PTH values, all favored a benign etiology of PHPT rather than malignancy-induced hypercalcemia. In addition, although she had a history of breast cancer, hypercalcemia of malignancy was unlikely as she was successfully treated for this diagnosis and there was no evidence of disease [5, 8]. Furthermore, the elevated 24-hour urinary calcium and absence of family history ruled out familial hypocalciuric hypercalcemia as a diagnosis. Last, the diagnosis of PHPT was further supported by the resolution of hypercalcemia following parathyroidectomy.

Parathyroidectomy is curative in most cases, and should be recommended to all patients who are surgical candidates and meet any of the surgical criteria: (1) serum calcium > 1 mg/dL (SI: 0.25 mmol/L) above the upper limit of normal; (2) skeletal involvement (fracture or bone mineral density T score below −2.5); (3) renal involvement (estimated glomerular filtration rate < 60 mL/min or radiologic evidence of nephrocalcinosis/nephrolithiasis, or hypercalciuria greater than 250 mg/day (SI: 62.5 mmol/day) in women and 300 mg/day (SI: 75 mmol/day) in men); and (4) age younger than 50 years [6]. In addition, the surgical option should also be discussed with asymptomatic patients with mild PHPT who are at low surgical risk if they have access to an experienced parathyroid surgeon, as parathyroidectomy has been associated with improvement in the bone mineral density and quality of life in this group [9, 10].

Successful preoperative localization of the parathyroids is generally associated with higher cure rates and significantly lower complications, related to shorter operative time, less risk to the nearby structures, and less tissue scarring [6]. In our patient, a neck ultrasound and ^99m^ Tc-sestamibi SPECT/CT of the neck and upper mediastinum were unrevealing. A subsequent 4D CT scan was able to identify the 2 adenomas, both measuring approximately 0.5 × 0.2 × 0.3 cm. These findings highlight the strengths and limitations of different imaging modalities. Neck ultrasound, for instance, is highly operator dependent and can perform poorly in identifying lesions—especially ectopic ones—located behind esophageal or osseous structures. Although ^99m^ Tc-sestamibi SPECT/CT is the most sensitive method of localization typically used for this purpose (sensitivity of 91.1%), there are certain adenoma and patient characteristics that affect the true positive (TP) rate of this method [11]. Swanson et al found that parathyroid adenomas measuring 1.9 to 3.5 cm were more likely to have TP SPECT/CT scans than those measuring 0.3 to 1.8 cm (*P* < .001) [12]. As a molecular imaging method, SPECT/CT also lacks the high spatial resolution of CT or magnetic resonance imaging, which can make very small lesions more difficult to detect. Swanson et al also found that preoperative hypercalcemia (ionized calcium 1.49-1.72 mmol/L) was more likely to be associated with TP scans compared to borderline elevated ionized calcium levels of 1.27 to 1.48 mmol/L (*P* < .05), reference range (1.14-1.32 mmol/L) [12]. Furthermore, Zhu et al reported that adenomas measuring ≤1.3 cm yielded a significant decrease in the sensitivity of neck ultrasounds, whereas a PTH level of ≤252 pg/mL (SI ≤ 252 ng/L) decreased sensitivity of SPECT/CT [11]. In contrast, 4D CT provides high spatial resolution and captures the dynamic arterial enhancement and rapid washout pattern that many parathyroid adenomas demonstrate. However, not all adenomas display this classic pattern and overreliance on expected enhancement behavior can lead to missed or misclassified adenomas [13]. The present patient's small adenomas, ionized calcium of 1.35 mmol/L, and PTH of 27 pg/mL (SI: 27 ng/L) illustrate the importance of considering these 3 characteristics, along with the technical limitations of each imaging modality, when evaluating the sensitivity of ultrasound, SPECT/CT, and 4D CT for the detection of parathyroid adenomas.

Although 10% to 15% of PHPT cases are due to multiple parathyroid adenomas, the simultaneous presence of both orthotopic and ectopic adenomas is rare and limited to isolated case reports [1]. For instance, Ogus et al reported a case of double adenomas, a solid one in the inferior right thyroid lobe and a cystic one in the mediastinum [14]. Tzikos et al reported the first case of PHPT resulting from ipsilateral double adenomas consisting of an ectopic and supernumerary one on the right lower neck in connection to the carotid sheath, and a subcapsular one [15]. There are only a few cases reporting the presence of a parathyroid adenoma in or near ectopic thymic tissue. Kordahi et al reported a retropharyngeal parathyroid adenoma with adjacent thymic tissue [16], whereas Funk et al reported an intrathymic parathyroid adenoma in the lateral neck [17]. Nevertheless, to our knowledge, there are no reports of dual parathyroid adenomas consisting of one in an orthotopic location and one in ectopic thymic tissue.

The embryological basis for this presentation lies in the shared origin of the inferior parathyroid glands and thymus from the third pharyngeal pouch. During normal development, the thymus migrates caudally into the anterior mediastinum, dragging the inferior parathyroids along their course. Variations in this migration process can result in ectopic positioning of either structure. Ectopic parathyroid tissue is most commonly located in the thymus, but in this case, the thymus itself was ectopically located in the thyroid bed, making this an exceptional anomaly.

The locations of these adenomas reinforce the importance of considering ectopic gland locations, including within the thymus when imaging studies are inconclusive but biochemical evidence supports the diagnosis of PHPT.

## Learning points

- Primary hyperparathyroidism can be caused by concomitant dual parathyroid adenomas, highlighting the importance of preoperative localizing studies.
- Parathyroid adenomas may present in atypical locations including embedded in ectopic or orthotopic thymic tissue.
- While the advances in imaging technology like 4D CT improve the sensitivity of preoperative parathyroid localization and are helpful for surgeons to achieve a successful parathyroid surgery, the intraoperative localization skill by surgical acumen remains indispensable.

## Data Availability

Original data generated and analyzed for this case report are included in this published article.
